# Precision medicine in acute lymphoblastic leukemia: A narrative review of current strategies and future directions

**DOI:** 10.1177/20406207261495830

**Published:** 2026-10-01

**Authors:** Sondus Jawad, Maria Benkhadra, Giuseppe Saglio, Rola Ghasoub, Mohamed A. Yassin

**Affiliations:** 1 College of Pharmacy, 61780 Qatar University, Doha, Qatar; 2 Department of Pharmacy, National Center for Cancer Care and Research, 36977 Hamad Medical Corporation, Doha, Qatar; 3 Department of Clinical and Biological Sciences, 9314 University of Turin, Turin, Italy; 4 Department of Hematology and Bone Marrow Transplant, 62850 National Center for Cancer Care and Research, Doha, Qatar

**Keywords:** Acute lymphoblastic leukemia, precision medicine, pharmacogenomics, targeted therapy, immunotherapy, molecular diagnostics

## Abstract

Acute lymphoblastic leukemia (ALL) is a genetically heterogeneous hematologic malignancy and the most common cancer in children, with significant morbidity and mortality in adults. Advances in molecular diagnostics and targeted therapies have shifted treatment from uniform chemotherapy toward precision medicine approaches tailored to individual genomic and clinical profiles. To summarize these approaches, a narrative review was conducted following the Scale for the Assessment of Narrative Review Articles (SANRA). A comprehensive literature search was performed in PubMed/MEDLINE, Embase, Scopus, and Web of Science for studies published between January 2015 and August 2026. Additional sources, including reference lists and clinical trial registries, were screened. Studies were selected based on relevance to precision medicine in ALL, including molecular diagnostics, pharmacogenomics, targeted therapy, and immunotherapy. Evidence was synthesized narratively without formal quality scoring. Key findings indicate that advances in next-generation sequencing have enabled the identification of clinically relevant molecular subtypes, including BCR-ABL1–positive, Philadelphia chromosome–like, and KMT2A-rearranged ALL, facilitating risk stratification and targeted treatment selection. Pharmacogenomic testing, particularly for TPMT and NUDT15 variants, supports individualized thiopurine dosing and reduces treatment-related toxicity. Targeted therapies and immunotherapies, including tyrosine kinase inhibitors, blinatumomab, inotuzumab ozogamicin, chimeric antigen receptor (CAR) T-cell therapy, and menin inhibition with revumenib for selected relapsed or refractory KMT2A-rearranged acute leukemias, have significantly improved outcomes in relapsed and refractory ALL. However, variability in access to molecular diagnostics, lack of standardized biomarker panels, and limited long-term outcomes data remain key challenges. Overall, Precision medicine is transforming the management of ALL by enabling individualized, biomarker-driven treatment strategies that improve survival and reduce toxicity. Broader implementation will require standardized diagnostic approaches, improved access to advanced therapies, and integration of real-world evidence to support clinical decision-making.

## Plain language summary

This narrative review explores recent advances in precision medicine for acute lymphoblastic leukemia (ALL), emphasizing genomic, pharmacogenomic, and immunotherapeutic innovations. ALL remains the most common childhood cancer and a major cause of adult leukemia mortality despite improved chemotherapy outcomes. The review highlights the role of next-generation sequencing in identifying key molecular subtypes, such as Ph-like and KMT2A-rearranged ALL, that influence prognosis and guide therapy selection. Pharmacogenomic testing for TPMT and NUDT15 variants enables safer thiopurine dosing and minimizes treatment-related toxicity. Targeted therapies and immunotherapies, including blinatumomab, inotuzumab ozogamicin, CAR T-cells, and emerging menin inhibitors like revumenib, have transformed treatment for relapsed and refractory cases. The review also discusses the integration of personalized thrombosis management based on genetic risk and anticoagulant metabolism. Despite these advances, disparities in access to molecular diagnostics and high costs limit global implementation. Future directions include multi-omics approaches and AI-based predictive models to optimize individualized care. Overall, precision medicine offers a pathway toward improved survival, reduced toxicity, and more equitable outcomes in ALL.

## 1. Introduction

Acute lymphoblastic leukemia (ALL) is the most common childhood malignancy and a major cause of cancer-related mortality in both pediatric and adult populations. Globally, ALL accounts for approximately 25% of childhood cancers, with peak incidence between two and five years of age.^1^ Advances in risk-adapted chemotherapy have improved pediatric cure rates to over 85% in high-income countries; however, outcomes in adults and in resource-limited settings remain suboptimal.^2^ Despite these successes, relapse, treatment-related toxicity, and variability in therapeutic response persist as significant clinical challenges.^3^ ALL is genetically heterogeneous, comprising distinct molecular subtypes characterized by chromosomal translocations, gene fusions, and sequence variants, each with prognostic and therapeutic implications.^4^ This heterogeneity underscores the need for therapeutic approaches tailored to the molecular and clinical profile of individual patients rather than a uniform treatment paradigm.

Over the past two decades, molecular profiling and pharmacogenomics have identified numerous biomarkers that influence prognosis and guide therapy. For example, thiopurine S-methyltransferase (TPMT) and nudix hydrolase 15 (NUDT15) polymorphisms significantly impact thiopurine metabolism and toxicity risk,^5^ while the identification of BCR-ABL1–positive ALL has enabled targeted treatment with tyrosine kinase inhibitors, transforming a historically poor-prognosis subgroup.^6^ Nevertheless, translating such genomic insights into widespread clinical application remains inconsistent.^7^ Some biomarkers are routinely integrated into treatment protocols, whereas others, despite strong supporting evidence, face adoption barriers related to cost, infrastructure, and standardization.^8^ Meanwhile, immunotherapies such as monoclonal antibodies, bispecific T-cell engagers like blinatumomab, and chimeric antigen receptor (CAR) T-cell therapy have expanded precision treatment options. Yet, questions remain regarding optimal sequencing, patient selection, long-term safety, and economic feasibility.^9^

The field of precision medicine in ALL is at an inflection point. Rapid advances in next-generation sequencing (NGS), multi-omics integration, and artificial intelligence–based analytics are generating unprecedented insights into disease biology and therapeutic vulnerabilities.^10^ Concurrently, new challenges have emerged, including ethical considerations in genomic data handling and inequities in access to high-cost targeted therapies. While systematic reviews excel at addressing narrow clinical questions, narrative reviews remain well-suited to synthesizing broad, rapidly evolving topics such as precision medicine in ALL.^11^ High-quality narrative reviews—assessed using tools such as the Scale for the Assessment of Narrative Review Articles (SANRA)—can critically integrate evidence across subfields, identify knowledge gaps, and propose strategic directions for both clinical practice and research.^12^

This review aims to provide an updated synthesis of current strategies in precision medicine for ALL, encompassing molecular diagnostics, pharmacogenomics, targeted therapies, and immunotherapies. It will critically appraise existing literature, outline key challenges, and explore future directions, bridging the gap between genomic discovery and real-world implementation to support more effective, individualized, and equitable care for patients with ALL.

## 2. Methods

This narrative review was conducted to synthesize current evidence on precision medicine approaches in acute lymphoblastic leukemia (ALL), including molecular diagnostics, pharmacogenomics, targeted therapies, and immunotherapies. The methodology was guided by the Scale for the Assessment of Narrative Review Articles (SANRA) to ensure transparency, structure, and methodological rigor in line with best practices for narrative reviews.^11,12^

### 2.1. Literature Search Strategy

A comprehensive literature search was performed across PubMed/MEDLINE, Embase, Scopus, and Web of Science to identify relevant studies published between January 2015 and August 2026, reflecting contemporary advances in precision medicine for ALL. To enhance coverage, Google Scholar and clinical trial registries such as ClinicalTrials.gov were also screened for grey literature and ongoing or recently completed studies. The search strategy combined controlled vocabulary (e.g., MeSH and Emtree terms) with free-text keywords. A representative search string applied in PubMed was as follows: (“Acute Lymphoblastic Leukemia” [Mesh] OR “ALL” OR “lymphoblastic leukemia”) AND (“precision medicine” OR “personalized medicine” OR “individualized therapy”) AND (“molecular diagnostics” OR “pharmacogenomics” OR “genomics” OR “targeted therapy” OR “immunotherapy” OR “CAR-T″ OR “bispecific antibody” OR “menin inhibitor” OR “KMT2A″ OR “blinatumomab” OR “ponatinib” OR “asciminib” OR “revumenib' OR “Ph-like” OR “ABL-class” OR “JAK-STAT”). Search strategies were adapted for each database, using Boolean operators (“AND”, “OR”), truncation, and phrase searching to maximize sensitivity and specificity.

### 2.2. Study Selection and Eligibility Criteria

Studies were eligible for inclusion if they were peer-reviewed original research articles, clinical trials, cohort studies, or high-quality narrative or systematic reviews; addressed precision medicine in ALL, encompassing molecular subtypes, pharmacogenomics, targeted therapy, or immunotherapy; involved pediatric, adolescent, or adult ALL populations; and were published in English between January 2015 and August 2026.

Studies were excluded if they were case reports, conference abstracts without complete data, editorials, or letters to the editor (unless containing substantial original data or expert consensus); were not directly related to ALL or precision medicine principles; or were published in languages other than English. Reference lists of included studies and relevant reviews were manually screened to identify additional eligible articles (backward citation tracking).

### 2.3. Data Extraction and Narrative Synthesis

Data were extracted and synthesized narratively, focusing on key domains of precision medicine in ALL, including molecular classification, pharmacogenomics, targeted therapies, and immunotherapy. Emphasis was placed on clinically relevant findings, consistency across studies, and applicability to real-world clinical practice. In cases of conflicting evidence between studies, priority was given to large randomized controlled trials, landmark studies, and publications with longer follow-up durations, while discrepancies were critically interpreted in the context of study design, population characteristics, and therapeutic settings. This approach aligns with best practices for narrative synthesis in rapidly evolving fields Evidence Synthesis.^11,12^

### 2.4. Quality Assessment

Consistent with the methodological framework of narrative reviews, no formal risk-of-bias assessment or quantitative quality scoring (e.g., GRADE or AMSTAR 2) was performed. Instead, study quality was evaluated qualitatively based on study design, sample size, methodological robustness, and relevance to precision medicine in ALL. The overall review process adhered to SANRA principles to ensure a balanced, comprehensive, and critical synthesis of the literature SANRA Scale.^12^

## 3. Results

### 3.1. Molecular Landscape of ALL

ALL is characterized by substantial molecular heterogeneity, with distinct genomic subtypes influencing prognosis and therapeutic responsiveness.^3,4^ Advances in NGS and transcriptomic profiling have identified recurrent chromosomal translocations, fusion genes, and somatic mutations that underpin leukemogenesis.^4,13^ Subtypes such as BCR-ABL1–positive ALL, ETV6-RUNX1–rearranged ALL, and hypodiploid ALL carry well-defined prognostic implications.^4,6^ The identification of “Ph-like” ALL—sharing a gene expression profile with BCR-ABL1–positive ALL but lacking the canonical fusion—has expanded molecular risk stratification frameworks and is associated with poor prognosis, as demonstrated in early landmark studies^13–16^ including Boer et al., who described the poor prognostic impact of this subtype.^16^

Molecular profiling not only refines risk classification but also guides therapeutic intensity. For example, IKZF1 deletions have been linked to poor prognosis and increased relapse risk, prompting consideration of augmented regimens.^4^ However, implementation barriers remain, including variable access to comprehensive genomic diagnostics and the need for international consensus on standard molecular panels. Clinically, these molecular insights have shifted treatment from purely morphology-based to genomically informed, enabling earlier identification of high-risk patients who may benefit from novel targeted agents.^13^ The major molecular subtypes of ALL are illustrated in Figure 1.

**Figure 1.**
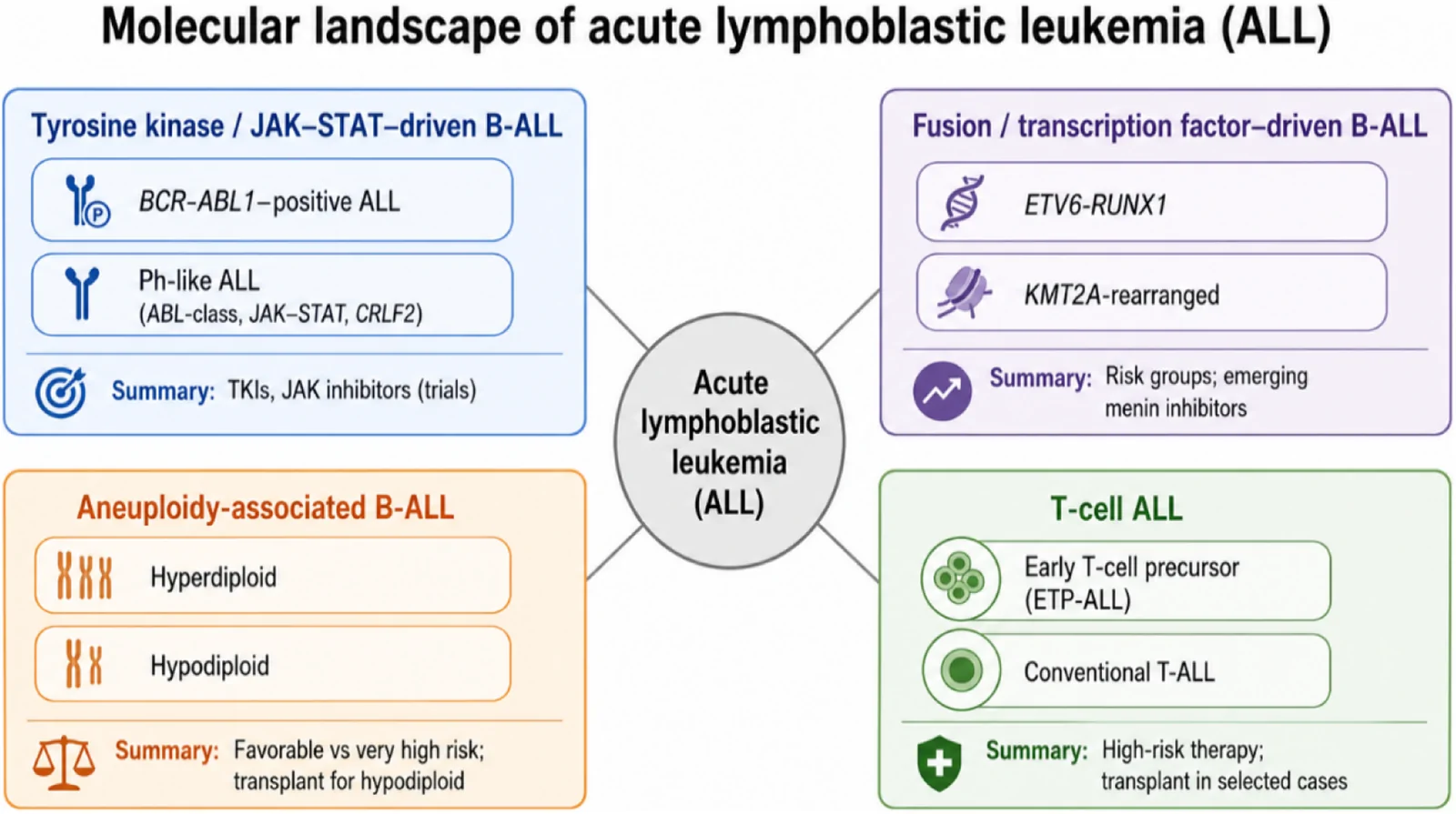
Molecular landscape of acute lymphoblastic leukemia (ALL). Conceptual map summarizing the major genomic subgroups of ALL, grouped into four broad clusters: tyrosine kinase/JAK–STAT–driven B-cell ALL (including BCR-ABL1–positive and Ph-like ALL with ABL-class, JAK–STAT, and CRLF2 lesions), aneuploidy-associated B-cell ALL (hyperdiploid and hypodiploid), fusion/transcription factor–driven B-cell ALL (ETV6-RUNX1 and KMT2A-rearranged), and T-cell ALL (early T-cell precursor and conventional T-ALL). Each cluster is annotated with representative precision-medicine implications, such as use of tyrosine kinase or JAK inhibitors, risk-adapted chemotherapy, emerging menin inhibitors, and hematopoietic stem cell transplantation in selected high-risk subgroups.

### 3.2. Pharmacogenomics in ALL

Pharmacogenomics has emerged as a cornerstone of precision medicine in ALL, focusing on germline genetic variants that influence drug metabolism, efficacy, and toxicity.^5,7^ The thiopurine S-methyltransferase (TPMT) and nudix hydrolase 15 (NUDT15) polymorphisms are the most clinically relevant markers, strongly associated with increased risk of myelosuppression during 6-mercaptopurine therapy.^5,7,17^ Routine genotyping for these variants allows dose individualization, reducing the likelihood of severe toxicity without compromising antileukemic efficacy.^5^

In addition to thiopurine metabolism, emerging evidence links genetic polymorphisms in folate pathway genes (e.g., MTHFR) and drug transporters (e.g., SLCO1B1) to methotrexate toxicity and clearance.^17,18^ Although these associations are biologically plausible, their clinical translation is limited by inconsistent replication across populations and study designs. Notably, population-based studies—such as those conducted in Latin America—highlight ethnic differences in the prevalence of pharmacogenomic variants, underscoring the need for population-specific dosing guidelines.^7^ Integration of pharmacogenomic testing into frontline care can reduce treatment-related morbidity, improve adherence, and optimize cure rates, but this requires coordinated infrastructure and reimbursement policies. Key pharmacogenomic markers and their clinical implications in ALL are summarized in Table 1, which has been expanded in this revision to include CPIC guideline recommendations and ethnic differences in variant prevalence (e.g., higher NUDT15*3 frequency in East Asian and Hispanic populations).

**Table 1.** Pharmacogenomic Markers and Clinical Implications in Acute Lymphoblastic Leukemia.

Gene	Drug	Clinical Impact	Clinical Recommendation
TPMT^5,7,17^	6-mercaptopurine	Increased risk of myelosuppression	Dose reduction based on genotype
NUDT15 (5, 7, 16)	6-mercaptopurine	Severe toxicity, particularly in Asian populations	Genotype-guided dosing
SLCO1B1 (17)	Methotrexate	Altered drug clearance and increased toxicity risk	Therapeutic drug monitoring
MTHFR^18^	Methotrexate	Variable association with toxicity	Not routinely recommended for clinical decision-making

Abbreviations: TPMT, thiopurine S-methyltransferase; NUDT15, nudix hydrolase 15; SLCO1B1, solute carrier organic anion transporter family member 1B1; MTHFR, methylenetetrahydrofolate reductase.

### 3.3. Targeted Therapies and Immunotherapies

Targeted therapy has transformed the management of genetically defined ALL subtypes as summarized in Table 1. TKIs such as imatinib and dasatinib have significantly improved outcomes in BCR-ABL1–positive ALL, converting a historically poor prognosis into a more manageable disease.^6,13^ Ph-like ALL is molecularly heterogeneous, and TKI responsiveness should not be assumed uniformly across the subtype. A clinically important subset of Ph-like ALL harbors ABL-class fusions (involving ABL1, ABL2, CSF1R, or PDGFRB) that activate signaling analogous to BCR-ABL1, and ABL-class fusions may provide a rationale for treatment with ABL-directed TKIs such as imatinib, dasatinib, or ponatinib, although the strength of clinical evidence varies by fusion partner and patient population.^19^ A second major group carries JAK-STAT pathway alterations (commonly CRLF2 rearrangements with or without JAK2, IL7R, or EPOR mutations); JAK inhibitors such as ruxolitinib remain investigational in JAK-STAT-altered Ph-like ALL; early-phase studies have provided biologic and clinical signals of activity, but efficacy has not been established sufficiently to support routine use outside clinical trials.^20^ A smaller remaining subset carries other targetable fusions (e.g., NTRK1/2/3, FGFR1), where agent selection is guided by the specific fusion partner and is currently supported only by case reports.^21^ TKI selection in Ph-like ALL should therefore be individualized to the underlying kinase-activating lesion rather than treated as a single therapeutic decision. For relapsed/refractory B-cell ALL, monoclonal antibodies like inotuzumab ozogamicin and bispecific T-cell engagers such as blinatumomab have demonstrated high remission rates, even in heavily pretreated populations.^9,14^

CAR T-cell therapy targeting CD19 has emerged as a highly effective salvage option, producing durable remissions in some patients.^22,23^ However, challenges remain, including cytokine release syndrome, neurotoxicity, high cost, and limited availability. While clinical trials have established impressive short-term efficacy, long-term durability and optimal sequencing with other therapies remain areas of active investigation.^14,18^

Blinatumomab, a bispecific T-cell engager (BiTE) targeting CD19, has shown efficacy across multiple ALL contexts. In relapsed/refractory ALL, it achieves high remission rates and can serve as a bridge to hematopoietic stem cell transplantation.^24^ Importantly, blinatumomab is now recognized as a valuable therapy for measurable residual disease (MRD)-positive disease, improving survival outcomes compared to chemotherapy alone.^25^ Recent studies have also expanded its role into frontline and consolidation therapy, where it demonstrates benefit in both pediatric and adult cohorts when combined with chemotherapy.^25,26^ These findings are supported by pivotal clinical trials that have established blinatumomab as a key component of therapy in both relapsed/refractory and high-risk frontline settings.^24,27^ In infants with KMT2A-rearranged ALL—a subgroup with historically poor outcomes—recent prospective data from van der Sluis et al. (N Engl J Med. 2023) have demonstrated the feasibility and efficacy of incorporating blinatumomab into treatment regimens, with improved remission rates and acceptable toxicity profiles.^27^ More recently, chemotherapy-free regimens combining ponatinib with blinatumomab have achieved high rates of complete molecular response as frontline therapy for Philadelphia chromosome–positive ALL,^28^ with emerging asciminib-based combinations representing a further evolution of this approach.^29^

Recent evidence has also established a role for blinatumomab in selected frontline settings. In the ECOG-ACRIN E1910 trial, adults with newly diagnosed BCR::ABL1-negative B-cell precursor ALL who achieved measurable residual disease–negative remission and were eligible for consolidation experienced improved overall survival when blinatumomab was added to consolidation chemotherapy, with a 3-year overall survival of approximately 85% versus approximately 68% with chemotherapy alone (hazard ratio 0.41).^30^ These findings support incorporating blinatumomab into frontline consolidation for selected adults, although applicability to older patients, those with persistent measurable residual disease, and pediatric populations requires consideration of disease biology, treatment tolerance, and protocol-specific evidence.

For patients with relapsed or refractory ALL, novel targeted agents are being developed. Menin inhibitors such as revumenib (SNDX-5613) target the menin–KMT2A interaction implicated in KMT2A-rearranged leukemogenesis^31^ and have shown encouraging activity in KMT2A-rearranged leukemias, including ALL, with high remission rates in early-phase clinical trials.^32^ These findings highlight the potential of epigenetic targeting in precision medicine for ALL relapse, though further validation in randomized studies is needed. In November 2024, the U.S. Food and Drug Administration approved revumenib for adults and children aged 1 year and older with relapsed or refractory acute leukemia harboring a KMT2A translocation, including ALL.^33^

### 3.4. Emerging Technologies and Future Directions

Advances in multi-omics approaches—including genomics, proteomics, metabolomics, and pharmacotranscriptomics—are enabling deeper biological insights and more precise therapeutic targeting.^10^ Artificial intelligence (AI) and machine learning algorithms are increasingly applied to integrate multi-dimensional datasets, predicting treatment response and toxicity risk with higher accuracy than conventional methods.^10^

Wearable biosensors and real-time monitoring technologies are being explored for early detection of complications such as febrile neutropenia or cardiac toxicity, enabling proactive interventions. In the long term, personalized vaccines targeting patient-specific leukemic neoantigens represent a promising but experimental frontier.^10^

These innovations could further individualize ALL therapy, but their integration into clinical practice will require robust validation, cost-effectiveness analyses, and ethical frameworks to manage genomic data responsibly.

## 4. Discussion

This review highlights the transformative role of precision medicine in the management of ALL. The integration of molecular diagnostics, pharmacogenomics, and targeted therapies has shifted treatment paradigms from uniform, protocol-driven regimens toward individualized care pathways informed by genomic and clinical risk factors.^3,4,13,14^ Molecular subtyping not only enables refined prognostic stratification but also serves as a predictive tool for targeted treatment selection, as exemplified by the use of ABL-class TKIs in BCR-ABL1–positive ALL and in the ABL-class subset of Ph-like ALL, whereas JAK-STAT–altered Ph-like ALL remains reliant on investigational JAK-inhibitor–based approaches.^13,14^ Similarly, pharmacogenomic testing—particularly for TPMT and NUDT15 variants—has proven clinical value in reducing treatment-related toxicity without compromising efficacy.^5,7,17^ This review highlights the transition from descriptive genomics to actionable precision oncology in ALL.

Emerging immunotherapies, including monoclonal antibodies, bispecific T-cell engagers, and CAR T-cell therapies, have significantly expanded salvage options for relapsed or refractory ALL.^9,14,22,23^ These therapies demonstrate high remission rates but require careful patient selection, vigilant toxicity monitoring, and economic considerations before integration into frontline regimens. Advances in precision medicine have also expanded beyond genomics into integrated therapeutic strategies, including optimized use of targeted therapies and immunotherapy across different disease settings.

Collectively, these innovations underscore that precision medicine in ALL is not a singular intervention but a continuum of approaches spanning diagnosis, treatment, and supportive care. The interplay between genomic profiling, pharmacogenomics, and immunotherapy is beginning to reshape the therapeutic landscape, though practical integration remains uneven across clinical settings.

The application of precision medicine in ALL has several tangible implications for clinical practice. First, risk-adapted therapy informed by molecular diagnostics can minimize overtreatment in low-risk patients while intensifying therapy for those at higher relapse risk.^4,13^ Second, pharmacogenomic-guided dosing can reduce life-threatening toxicities, improve adherence, and potentially reduce treatment interruptions.^5,7,17^ Third, the integration of biomarker-guided immunotherapy offers new hope for patients with otherwise poor prognoses, potentially shifting some therapies earlier in the treatment algorithm.^14,22,23^

To translate these benefits broadly, clinicians must have access to rapid, cost-effective molecular and pharmacogenomic testing, alongside institutional pathways for acting on results. Multidisciplinary teams—including hematologists, molecular pathologists, pharmacists, and genetic counselors—will be central to successful implementation.

Several knowledge gaps emerged from this review (Table 2).

**1. Standardization of molecular testing**: While NGS-based profiling is increasingly available, there is no universal consensus on which biomarkers should be included in routine testing panels for ALL across age groups.^13^
**2. Population diversity in pharmacogenomics**: Most pharmacogenomic evidence comes from Caucasian and East Asian populations, with limited data from African, Middle Eastern, and Latin American cohorts, despite known differences in variant prevalence.^7,17^
**3. Long-term outcomes of immunotherapies**: Data on durability of remission, late toxicities, and secondary malignancies after CAR T-cell and bispecific antibody therapy remain limited.^22,23^
**4. Cost-effectiveness and health equity**: There is a lack of rigorous health economics research assessing the impact of precision medicine strategies on healthcare budgets and access in low- and middle-income countries.^10^

**Table 2.** Key Precision Medicine Strategies in Acute Lymphoblastic Leukemia.

Domain	Example	Clinical Impact
Genomics	Next-generation sequencing (NGS)	Risk stratification and subtype identification^4,6,13–16^
Pharmacogenomics	TPMT, NUDT15	Individualized dosing and reduced toxicity^5,7,17^
Targeted therapy	TKIs in BCR::ABL1-positive ALL and alteration-directed therapy in selected Ph-like ALL	TKIs improve outcomes in BCR-ABL1-positive ALL.^6,13^ In Ph-like ALL, treatment depends on the underlying actionable lesion: ABL-class fusions may support ABL-directed TKI therapy, whereas JAK-STAT alterations remain investigational treatment targets.^19,20^
Immunotherapy	CAR T-cell therapy, blinatumomab	High remission rates in relapsed/refractory ALL^9,14,22,23^
Emerging approaches	Multi-omics and artificial intelligence	Enhanced prediction of treatment response and personalization of care^10^

Addressing these gaps will require coordinated international research, including prospective multicenter trials and real-world registry studies.

## 5. Limitations

This narrative review is subject to several limitations. Although a structured search strategy was applied across multiple databases, the methodology was not as exhaustive as that used in systematic reviews, which may introduce selection bias.^11,12^ Only English-language studies published between 2015 and 2026 were included, potentially excluding relevant findings in other languages or from earlier landmark studies. The qualitative nature of narrative synthesis also means that no formal meta-analytic assessment of effect sizes or heterogeneity was performed. Finally, while every effort was made to balance perspectives, the rapidly evolving nature of precision medicine in ALL means that emerging data published after the search cut-off may not be reflected. Additionally, potential underrepresentation of studies from low- and middle-income countries and non-Western populations may limit the generalizability of some conclusions, particularly regarding pharmacogenomic variant frequencies and access to novel therapies.

## 6. Conclusion

Precision medicine is rapidly transforming the diagnosis and treatment of ALL, moving the field beyond uniform chemotherapy protocols toward individualized, biomarker-guided strategies.^3,4,13,14^ Molecular subtyping now enables refined prognostic classification, while pharmacogenomic testing—particularly for TPMT and NUDT15 variants—allows for safer, more effective dosing of key agents such as thiopurines.^5,7,17^ Targeted agents, including ABL-class TKIs for BCR-ABL1–positive and ABL-class Ph-like ALL, emerging JAK-inhibitor–based strategies for JAK-STAT-altered Ph-like ALL, monoclonal antibodies, bispecific T-cell engagers, and CAR T-cell therapies, have expanded the therapeutic arsenal for high-risk and relapsed populations.^9,18,22,23^ In parallel, emerging precision medicine approaches continue to refine the use of targeted therapies and immunotherapies across different disease settings.

Despite these advances, challenges remain in ensuring equitable access, standardizing molecular testing panels, validating pharmacogenomic findings across diverse populations, and assessing the cost-effectiveness of high-cost targeted therapies.^10,26^ The integration of multi-omics approaches with artificial intelligence–driven predictive models holds promise for deeper disease characterization and real-time treatment optimization.^10^ However, their successful adoption will require robust infrastructure, clinician education, and ethical frameworks for genomic data management.

In the coming decade, the most impactful progress in ALL precision medicine will likely come from the integration of diagnostic genomics, pharmacogenomics, and novel targeted therapeutics into seamless, multidisciplinary care pathways. Achieving this vision will require international collaboration, real-world evidence generation, and sustained commitment to health equity—ensuring that the benefits of precision oncology are available to all patients, regardless of geography or socioeconomic status.
